# Supplementary material for: Sedimentary DNA tracks decadal-centennial changes in fish abundance
Source: Commun Biol. 2020 Oct 8;3:558. doi: 10.1038/s42003-020-01282-9 (PMC7546629; doi:10.1038/s42003-020-01282-9)
Supplement: Supplementary file 1 — Supplementary Information [file 42003_2020_1282_MOESM1_ESM.pdf]

Supplementary Information for Kuwae et al., entitled 'Sedimentary DNA tracks decadal-centennial changes in fish abundance'

Supplementary Figures

Supplementary Figure 1. Study area (a) and site of sediment core collection in Beppu Bay. Western Seto Inland Sea includes Beppu Bay, Iyo-nada, Suo-nada. Central Seto Inland Sea includes Hiroshima Bay, Aki-nada, and Hiuchi-nada. The Bungo Channel connect between the western Seto Inland Sea and the Pacific.

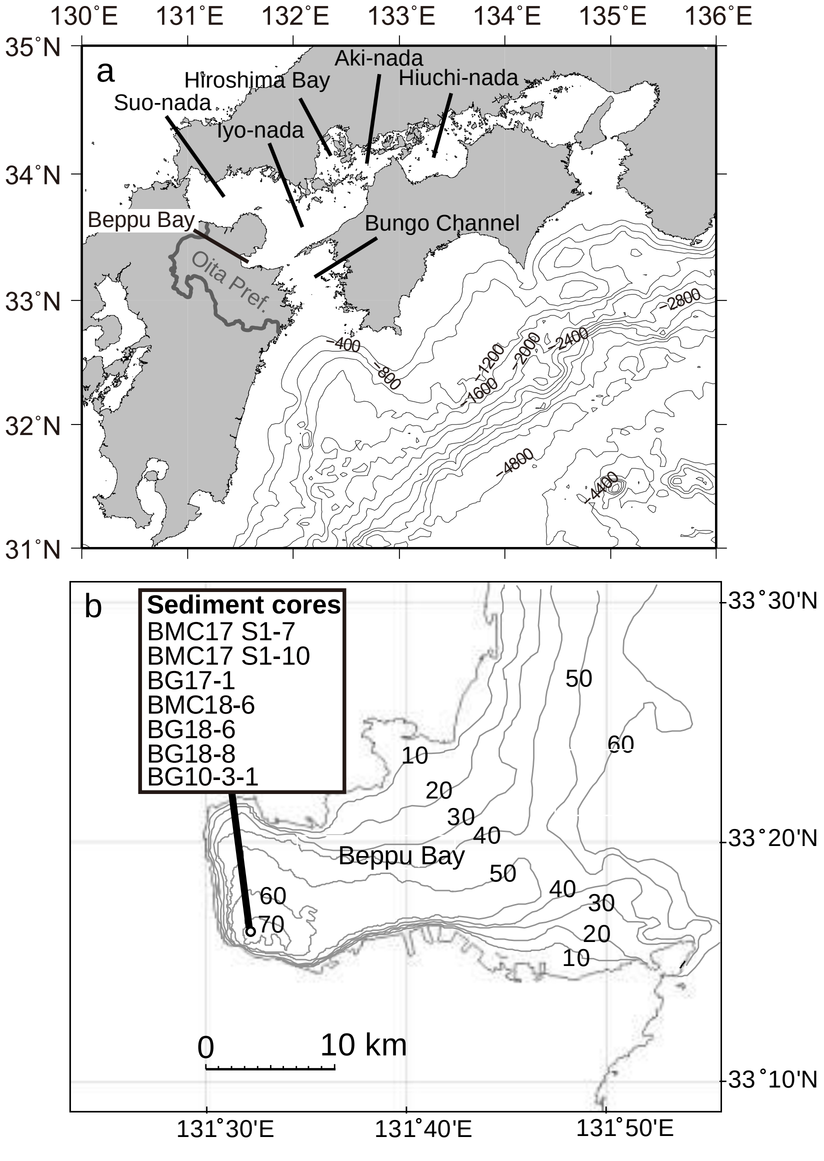

Supplementary Figure 2. Vertical distributions of DNA concentrations for each short core. a: *Engraulis japonics* (Japanese anchovy); b: *Sardinops melanostictus* (Japanese sardine); and c: *Tranchurus japonics* (jack mackerel). Error bar of each data point denotes 1SD.

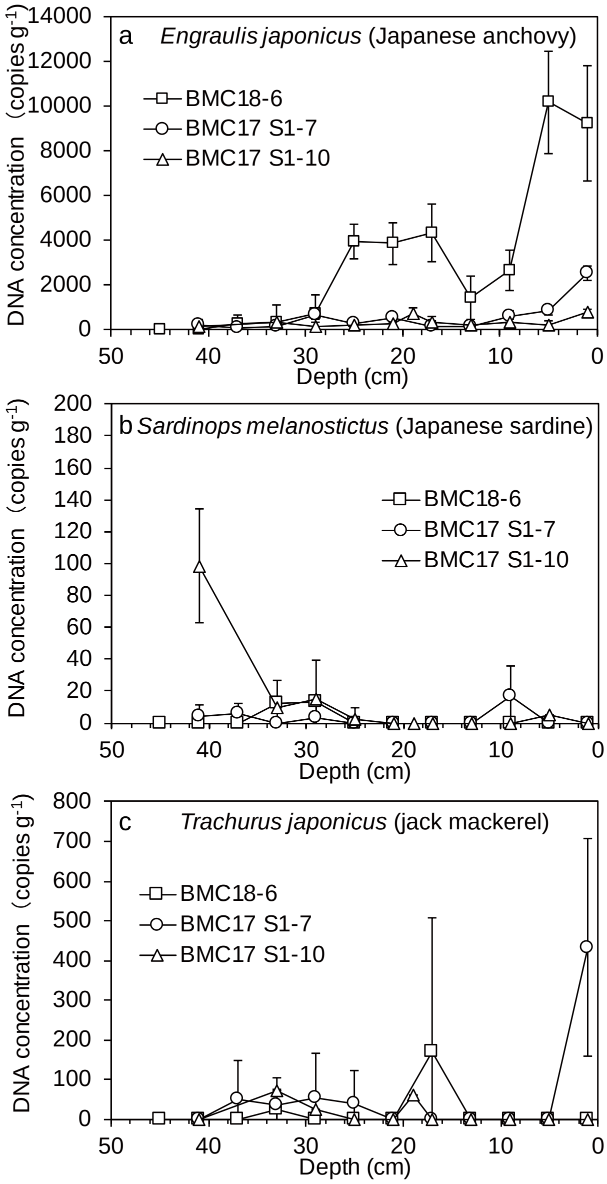

Supplementary Figure 3. Vertical distributions of DNA concentrations for each 1.2-m core. a: *Engraulis japonics* (Japanese anchovy); b: *Sardinops melanostictus* (Japanese sardine); and c: *Tranchurus japonics* (jack mackerel). Error bar of each data point denotes 1SD.

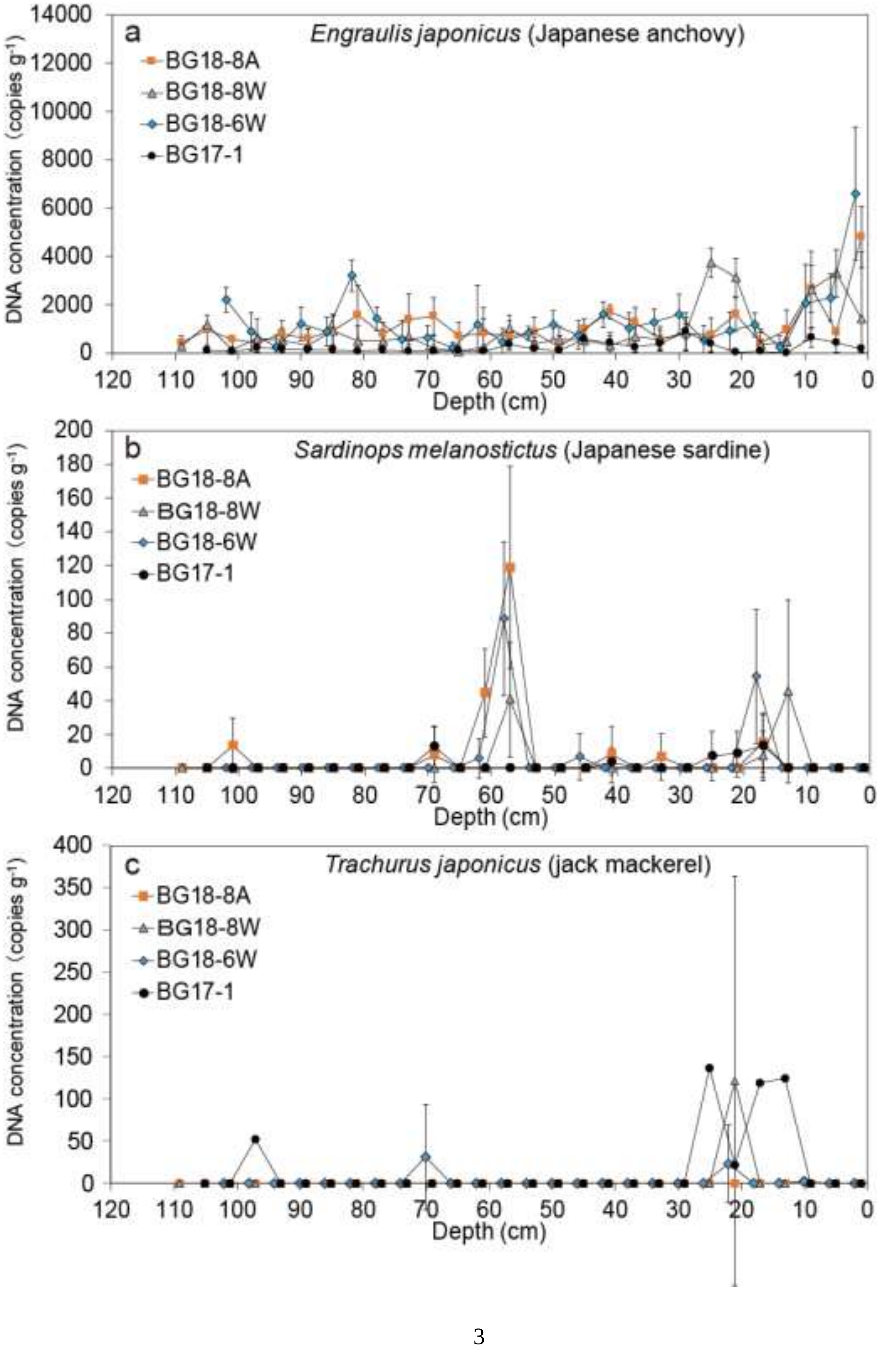

Supplementary Figure 4. Fish scale concentration of Japanese anchovy (a) and Japanese sardine (b) for each core previously reported in Kuwae et al. (2017).

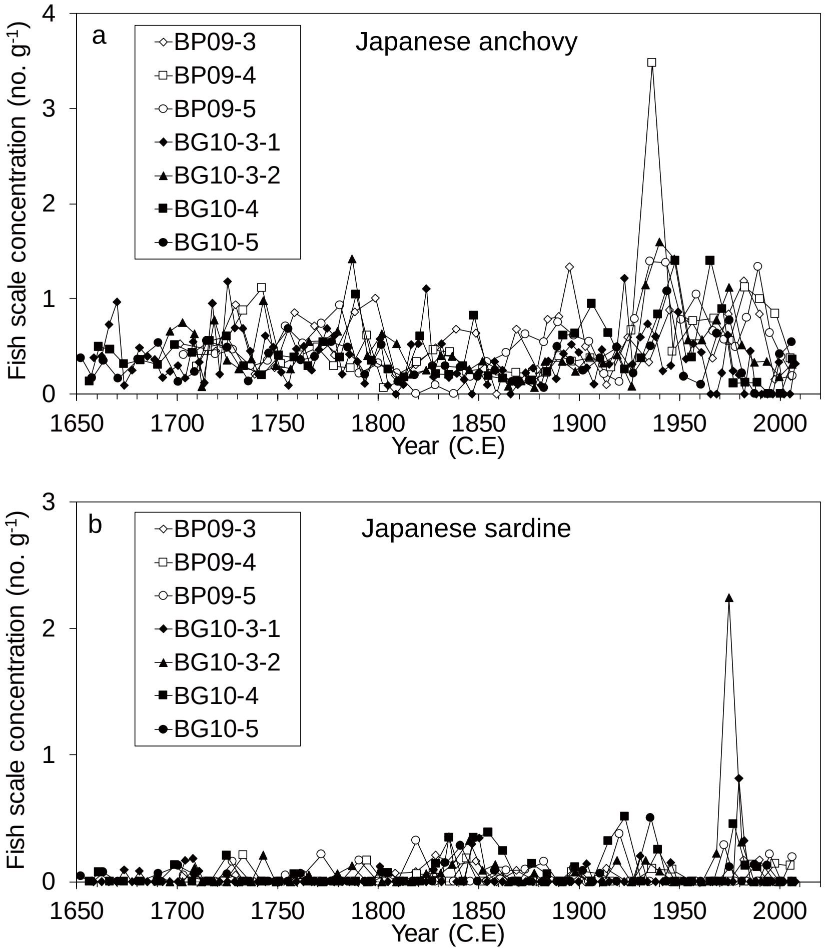

Supplementary Figure 5. Relationships between $\log_{10}$-transformed values of eDNA concentrations and fish scale concentrations for (a) Japanese anchovy, *Engraulis japonicus* and (b) Japanese sardine, *Sardinops melanostictus*. Inter-core, 7-yr average data were used for the models. The Type II regression model of $\log_{10}$-transformed values were performed; ($R^2 = 0.0146$, $P = 0.171$, $n = 42$ for a) and $R^2 = 0.578$, $P = 4.96\times10^{-9}$, $n = 42$ for b). Red line denotes a regression line of significant Type II regression model with the 95% confidence interval (gray zone).

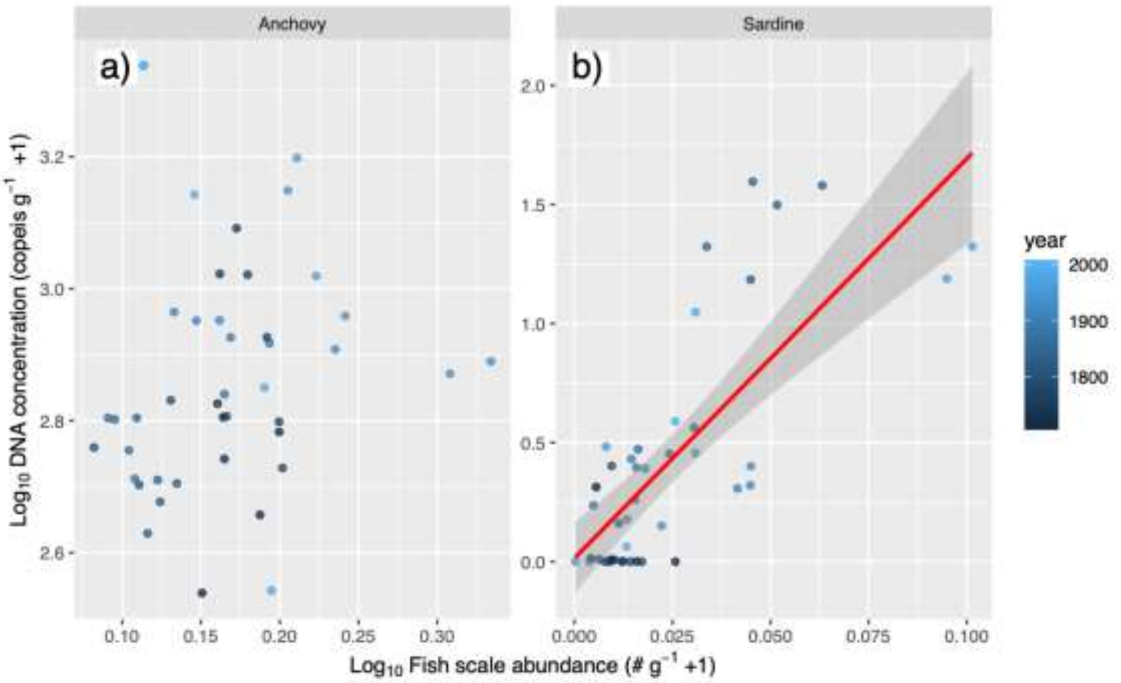

Supplementary Figure 6. Relationships between $\log_{10}$-transformed values of eDNA concentrations and the total landings for (a) Japanese anchovy, *Engraulis japonicus* and (b) Japanese sardine, *Sardinops melanostictus*, (c) jack mackerel, *Tranchurus japonicus*, and (d) *Caranginae* (jack mackerel and amberstripe scad). Inter-core, 7-yr average data were used for the models. The Type II regression model of $\log_{10}$-transformed values were performed; ($R^2 = 0.269$, $P = 0.031$, $n = 16$ for a) and $R^2 = 0.345$, $P = 0.016$, $n = 16$ for b), $R^2 = 0.067$, $P = 0.536$, $n = 8$ for c) and $R^2 = 0.677$ $P = 2.78\times10^{-5}$, $n = 18$ for d), Red line denotes a regression line of significant Type II regression model with the 95% confidence interval (gray zone).

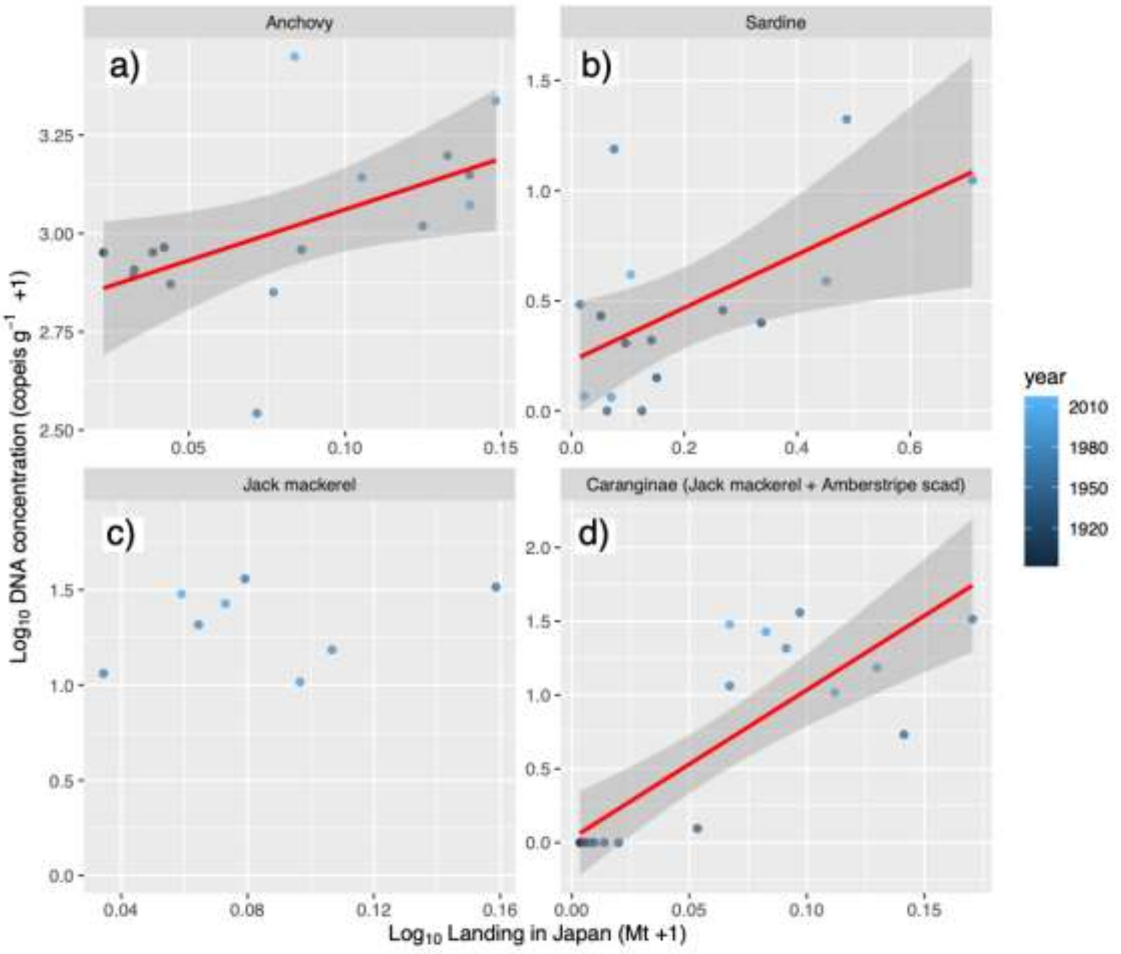

Supplementary Figure 7. Comparisons between anchovy landing from the Bungo Channel (a), Beppu Bay, Iyo-nada and Suo-nada (b), and the central Seto Inland Sea (c) and anchovy sedDNA (d) with the total anchovy landing in Japan. Left- and right-hand scale for the upper three panels denote the total landing in Japan and the local landing, respectively. Error bar of each data in (d) point denotes 1SD.

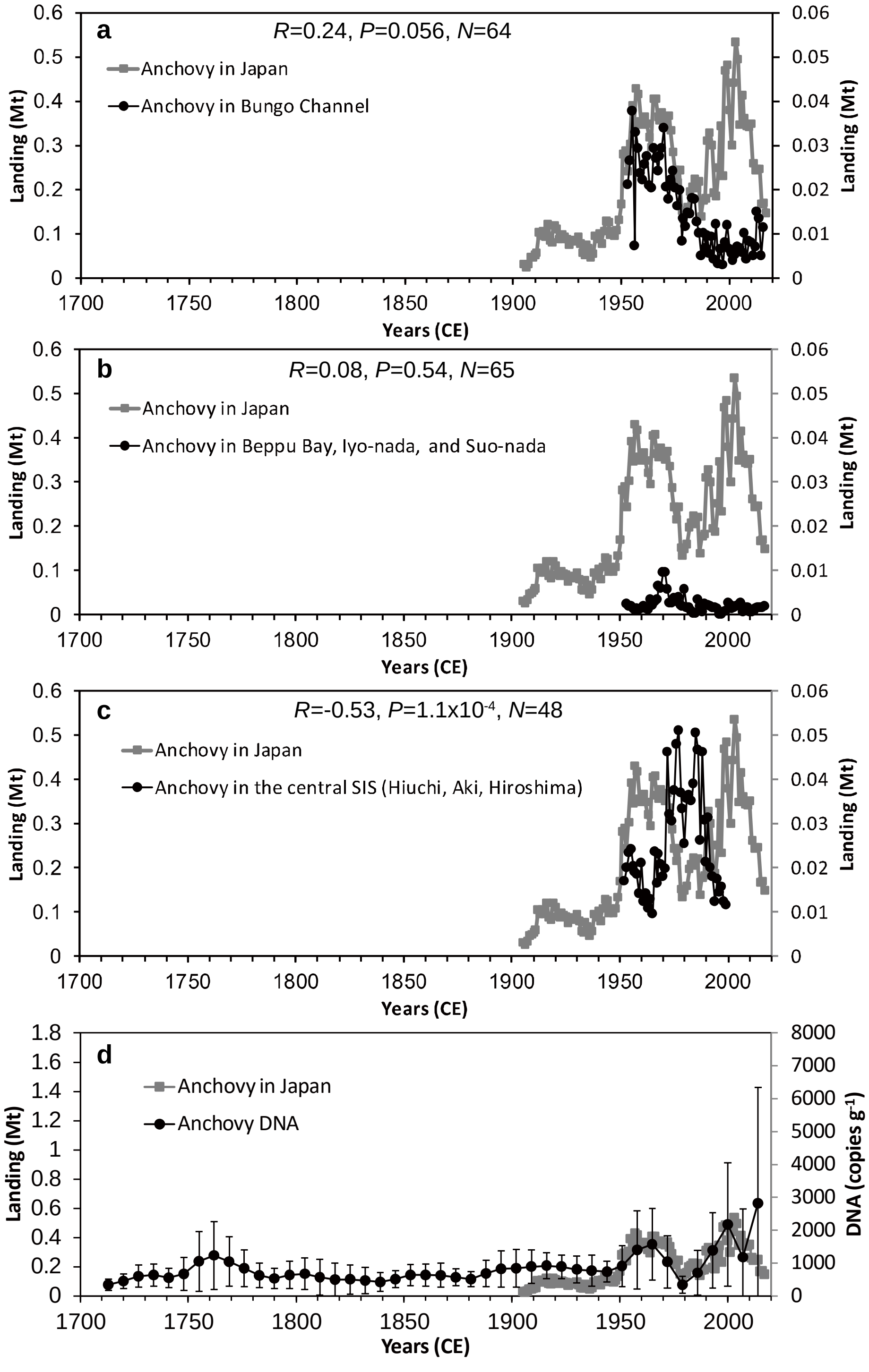

Supplementary Figure 8. Comparison between sardine landing from the Bungo Channel (a), Beppu Bay, Iyo-nada, and Suo-nada (b), and the central Seto Inland Sea (c) and sardine sedDNA (d) with the total sardine landing in Japan. Left- and right-hand scale for the upper three panels denote the total landing in Japan and the local landing, respectively. Error bar of each data in (d) point denotes 1SD.

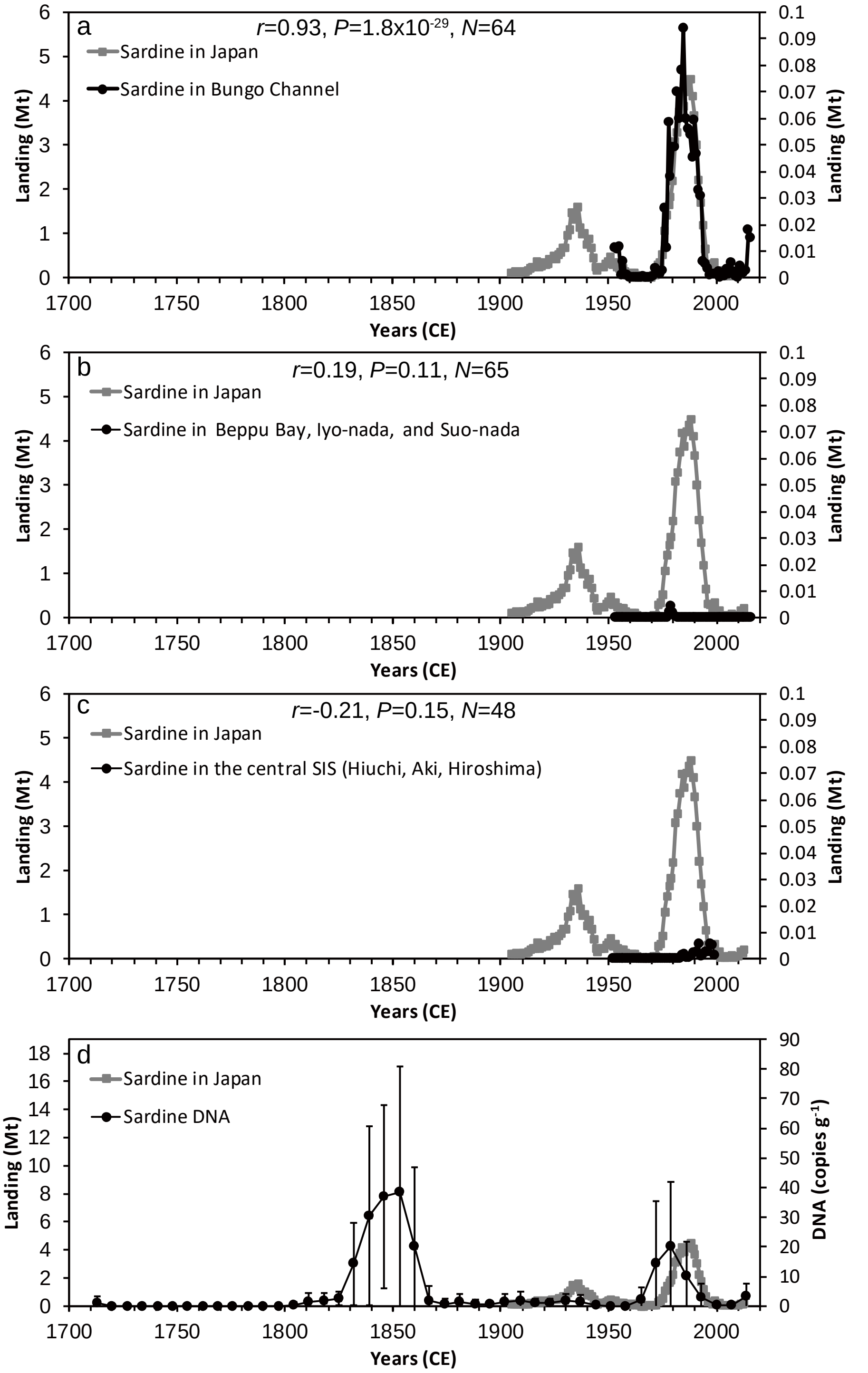

Supplementary Figure 9. Comparison between jack mackerel landing from the Bungo Channel (a), jack mackerel landing from Beppu Bay, Iyo-nada, and Suo-nada (b), and *Caranginae* (jack mackerel + Amberstripe scad) landing from the central Seto Inland Sea (c), and jack mackerel sedDNA with the total *Caranginae* landing in Japan. Left- and right-hand scale for the upper three panels denote the total landing in Japan and the local landing, respectively. Error bar of each data in (d) point denotes 1SD.

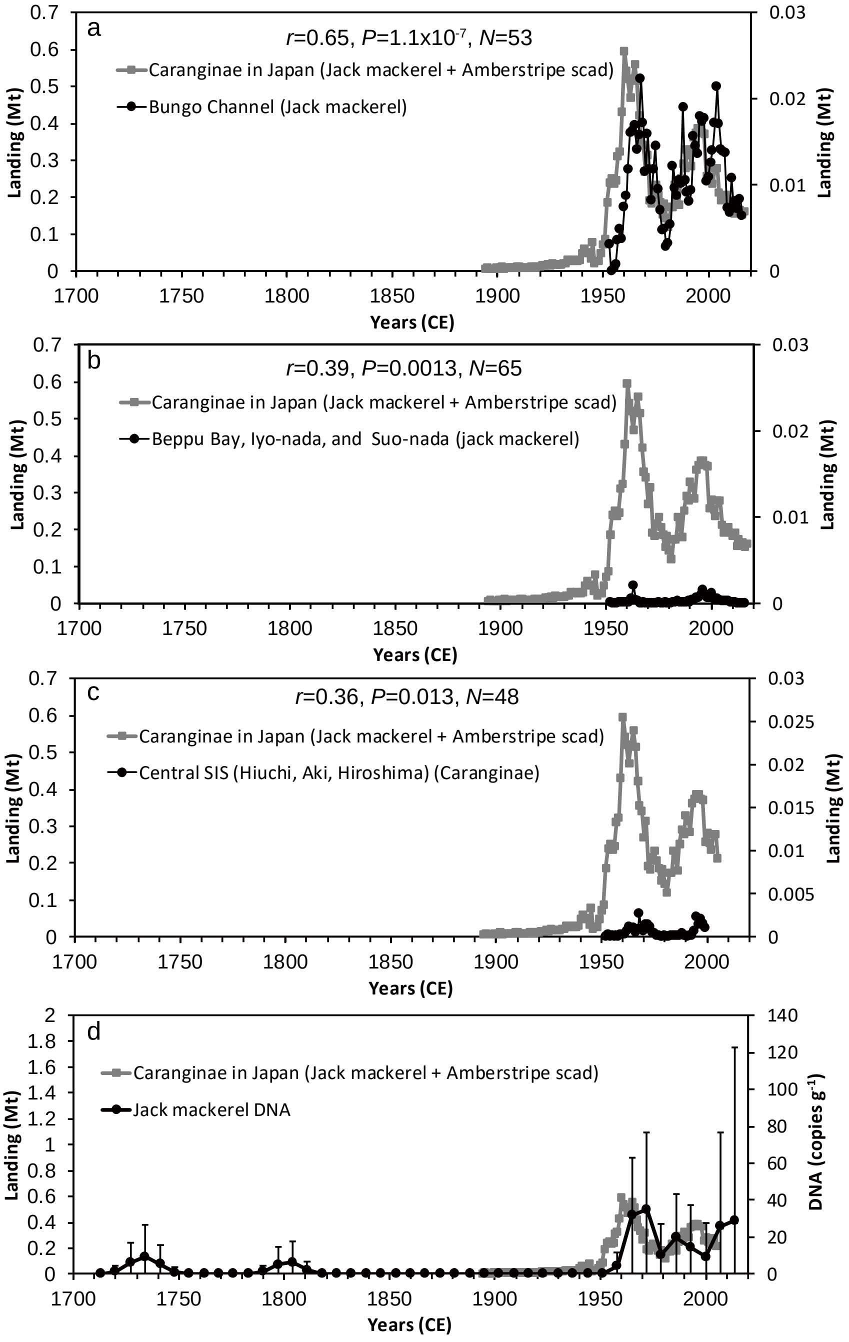

Supplementary Figure 10. Comparison between temporal variations in anchovy DNA and sedimentary properties.

Thin and bold line denote data from BG17-1 and BMC17 S1-10, respectively.

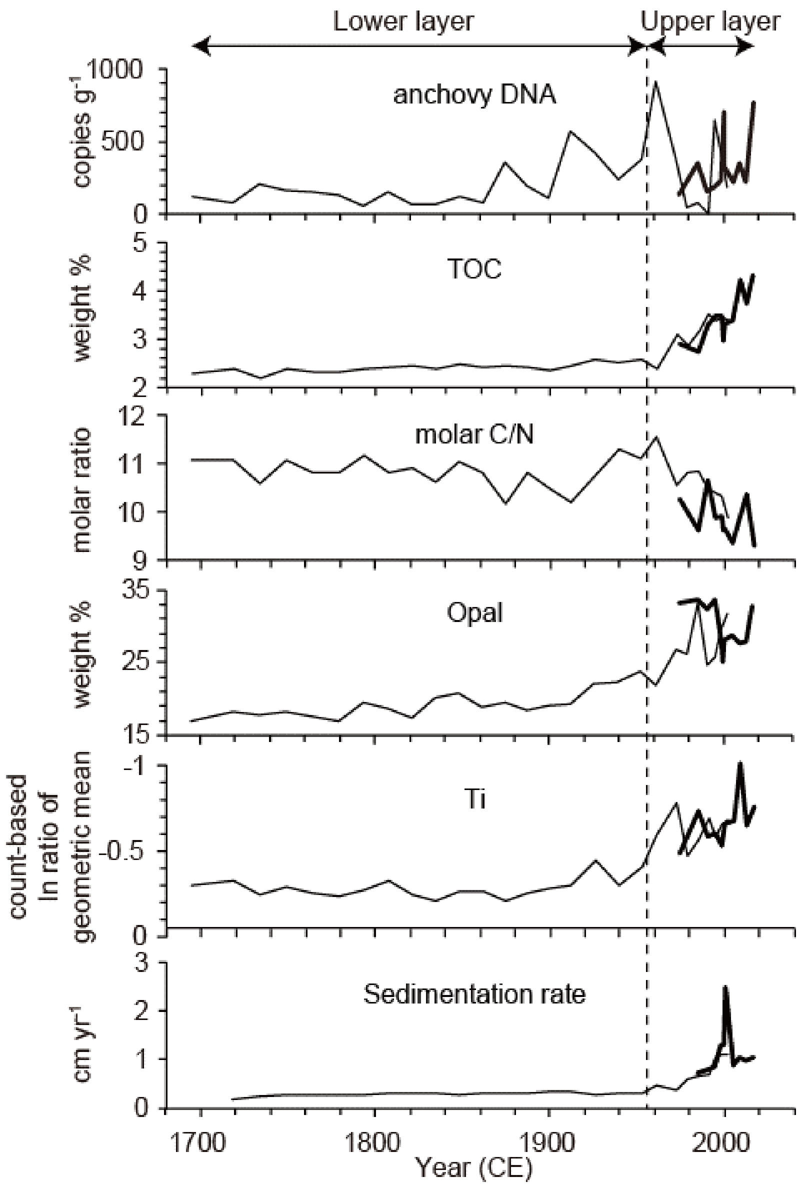

Supplementary Figure 11

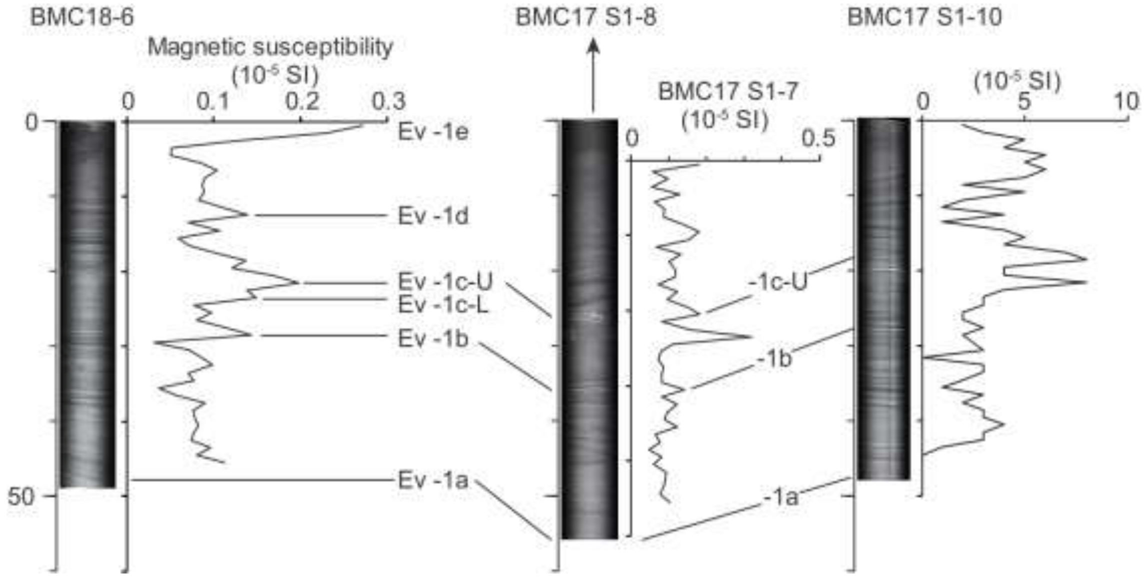

Supplementary Figure 12. Sediment core samples used in this study. Photo, CT image, and magnetic susceptibility for each core. Horizontal lines connected between the cores denote inter-core correlation between event layers characterized by high-density, high magnetic susceptibility.

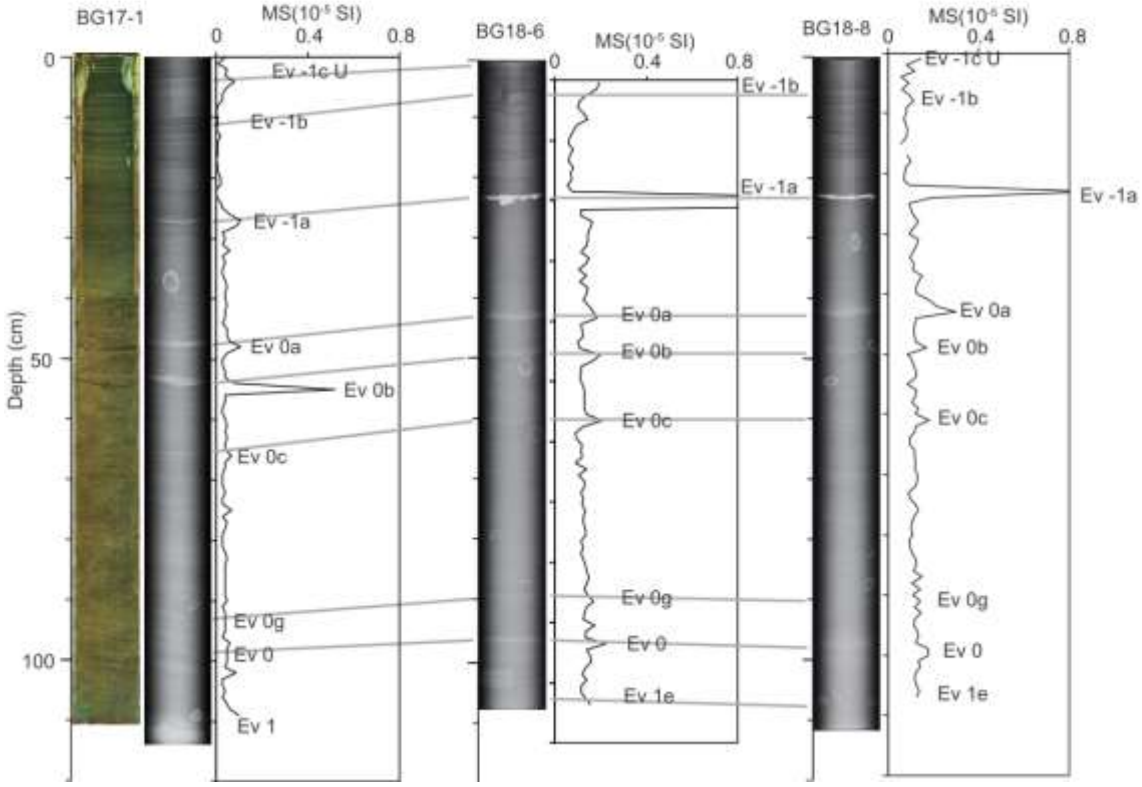

Supplementary Figure 13. Particles in the coarse size fraction (BHR17-1 16-17 cm 63–180µm ×40). Arrow denotes a pellet.

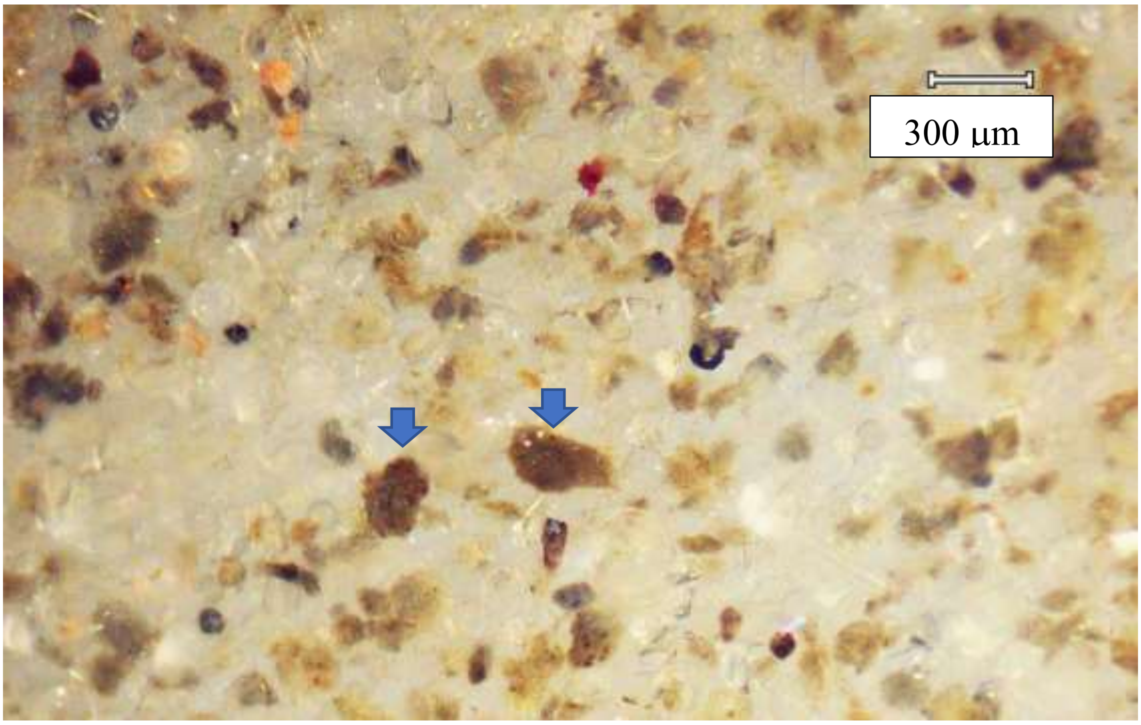

Supplementary Figure 14. Particles in the coarse size fraction (BHR17-1 20–21 cm 63–180μm ×20). Arrow denotes a pellet.

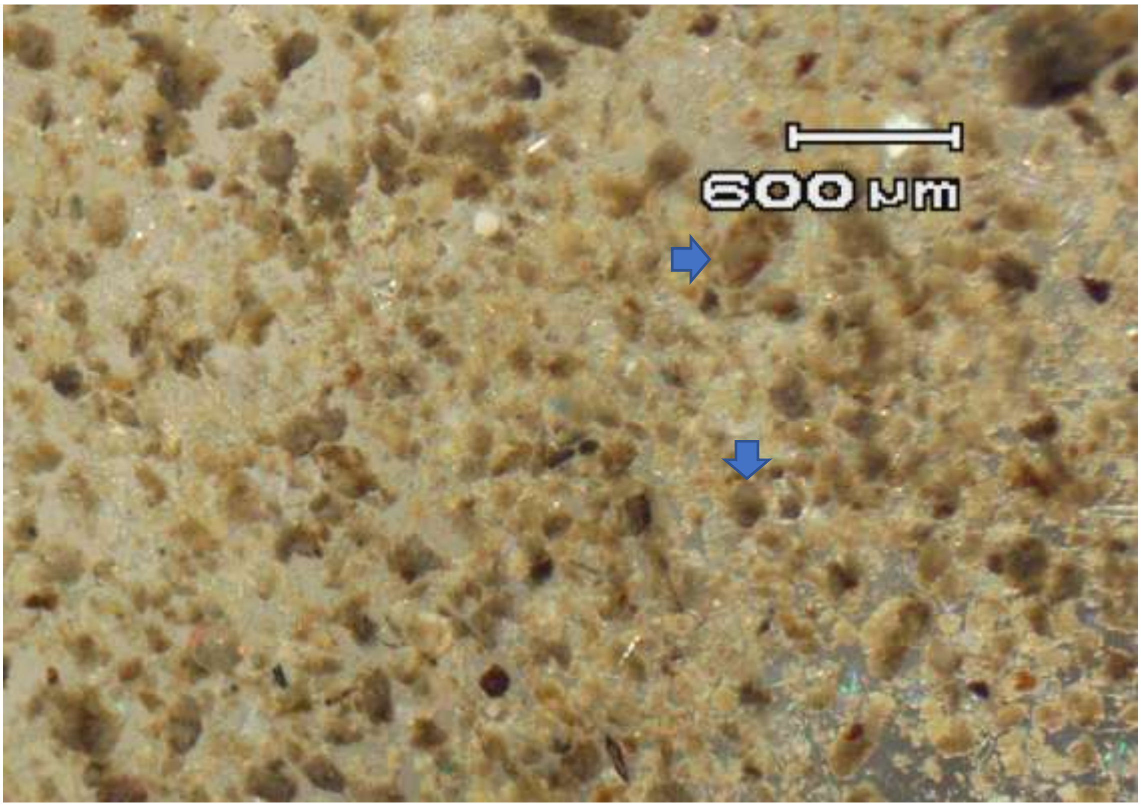

Supplementary Figure 15. Particles in the coarse size fraction (BHR17-1 16–17 cm 180–500μm ×40). Arrow denotes a pellet.

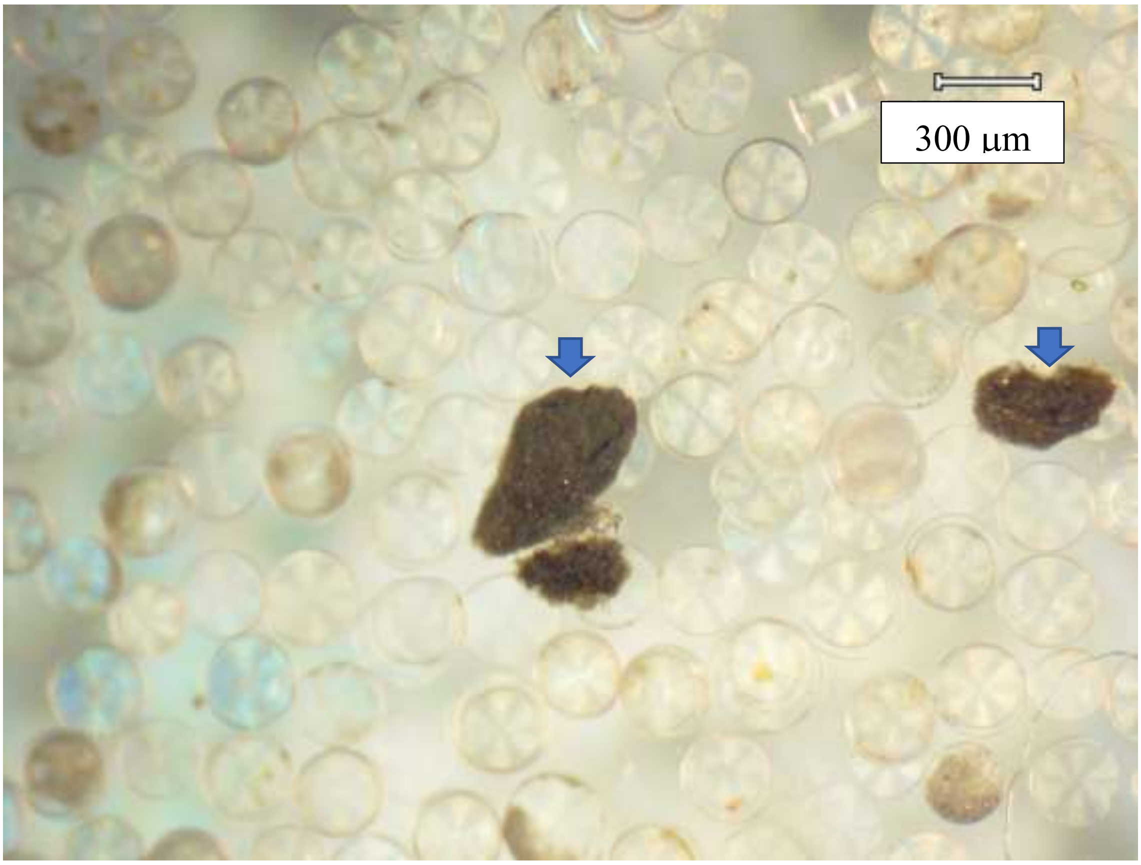

Supplementary Figure 16. Particles in the coarse size fraction (BHR17-1 25–26 cm 180–500µm ×20). Arrow denotes a pellet.

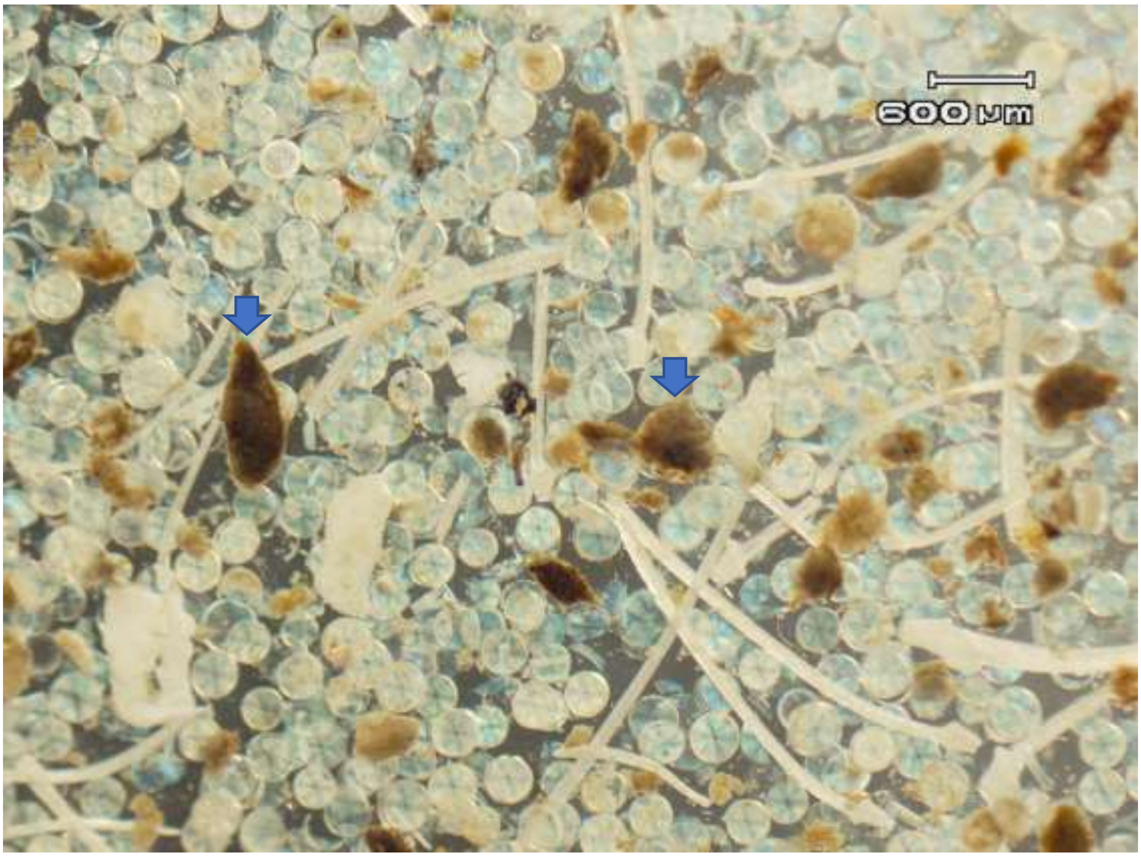

Supplementary Tables

Supplementary Table 1. Sediment samples and core depths for DNA analysis, mean and 1SD of DNA copies for each species for bulk sediment samples for each core, and the number of DNA detection against the total number of replicates for each core.

sample ID	Core depth top (cm)	Core depth bottom (cm)	bulk sediment mean (copies g^{-1} dry)	S.D.	detection/total replicates
Japanese anchovy					
BG17-1	0	105	233.9	215.2	92/128
BG18-6W	0	102	1359.7	1284.5	89/104
BG18-8W	0	109	940.5	1004.4	82/112
BG18-8A	0	109	961.9	519.2	93/112
BMC17 S1-7	0	41	558.6	705.0	39/52
BMC17 S1-10	0	41	340.3	213.2	36/44
BMC18-6	0	45	3075.5	781.9	34/48
7 cores average	-	-	1067.2	968.5	465/600
LBHR18-1	0	5	ND	-	0/4
blank	-	-	ND	-	0/4
Japanese sardine					
BG17-1	0	105	1.7	4.0	14/116
BG18-6W	0	102	6.0	20.0	9/103
BG18-8W	0	109	3.4	11.4	7/112
BG18-8A	0	109	8.0	24.0	15/112
BMC17 S1-7	0	41	2.7	5.1	7/44
BMC17 S1-10	0	45	12.0	29.2	10/44
BMC18-6	0	41	2.1	8.3	3/48
7 cores average	-	-	5.1	3.8	65/579
LBHR18-1	0	5	ND	-	0/4
blank	-	-	ND	-	0/4
Jack mackerel					
BG17-1	0	105	10.4	26.7	
BG18-6W	0	102	2.2	7.4	4/104
BG18-8W	0	109	4.3	22.9	1/112
BG18-8A	0	109	0.0	0.0	0/112
BMC17 S1-7	0	41	55.9	127.2	8/44
BMC17 S1-10	0	45	14.3	26.8	3/68
BMC18-6	0	41	16.2	97.7	2/48
7 cores average	-	-	14.8	19.1	18/488
LBHR18-1	0	5	ND	-	0/4
blank	-	-	ND	-	0/4

Supplementary Table 2. Results (ΔCt) of spike test for evaluating PCR inhibition effect. ΔCt value with less than three indicates no effect of enzymatic inhibition on PCR amplification for a sample.

BMC17 S1-7		BMC17 S1-10		BMC18-6W		BG17-1W		BG18-6W		BG18-8W		BG18-8A	
Depth (cm)	ΔCt	Depth (cm)	ΔCt	Depth (cm)	ΔCt	Depth (cm)	ΔCt	Depth (cm)	ΔCt	Depth (cm)	ΔCt	Depth (cm)	ΔCt
0-1	-0.2	0-1	-0.1	0-1	-1.8	0-1	-0.1	0-2	-1.3	0-1	-0.2	0-1	0.1
4-5	-0.1	4-5	-0.3	4-5	-2.2	4-5	0.0	4-6	-1.7	4-5	0.0	4-5	0.0
8-9	-0.1	8-9	-0.1	8-9	-2.0	8-9	0.0	8-10	-1.3	8-9	-0.1	8-9	-0.1
12-13	-0.3	12-13	-0.3	12-13	-1.7	12-13	0.1	12-14	-2.2	12-13	-0.2	12-13	0.0
16-17	0.0	16-17	-0.1	16-17	0.0	16-17	-0.1	16-18	-1.6	16-17	-0.1	16-17	0.0
20-21	-0.1	18-19	0.0	18-19	0.1	20-21	-0.2	20-22	-2.3	20-21	0.0	20-21	0.1
24-25	0.0	20-21	-0.2	20-21	0.1	24-25	0.0	24-26	-2.1	24-25	0.0	24-25	0.0
28-29	-0.2	24-25	-0.2	24-25	-0.1	28-29	-0.1	28-30	-1.6	28-29	-0.1	28-29	0.0
32-33	-0.2	28-29	0.0	28-29	0.0	32-33	0.1	32-34	-1.9	32-33	-0.1	32-33	0.0
36-37	-0.1	32-33	-0.1	32-33	0.0	36-37	-0.2	36-38	-2.4	36-37	-0.2	36-37	0.1
40-41	-0.1	40-41	-0.1	40-41	-0.1	40-41	-0.1	40-42	-1.3	40-41	-0.2	40-41	0.1
				44-45	0.0	44-45	-0.2	44-46	-2.1	44-45	0.0	44-45	-0.1
						48-49	-0.1	48-50	-2.0	48-49	0.0	48-49	0.0
						52-53	-0.3	52-54	-2.4	52-53	-0.1	52-53	0.3
						56-57	0.0	56-58	-1.4	56-57	-0.4	56-57	0.5
						60-61	-0.2	60-62	-2.1	60-61	-0.1	60-61	-0.1
						64-65	0.0	64-66	-1.9	64-65	0.0	64-65	0.0
						68-69	-0.3	68-70	-2.3	68-69	0.0	68-69	2.9
						72-73	-0.1	72-74	-1.8	72-73	0.0	72-73	0.1
						76-77	-0.1	76-78	-2.1	76-77	0.1	76-77	0.0
						80-81	-0.2	80-82	-2.0	80-81	-0.1	80-81	0.1
						84-85	-0.3	84-86	-2.2	84-85	0.2	84-85	0.5
						88-89	-0.3	88-90	-2.0	88-89	0.2	88-89	0.2
						92-93	-0.3	92-94	-2.2	92-93	0.0	92-93	0.5
						96-97	-0.2	96-98	-1.4	96-97	0.0	96-97	0.0
						100-101	0.1	100-102	-2.1	100-101	-0.1	100-101	0.0
						104-105	-0.2			104-105	-0.2	104-105	-0.1
										108-109	-0.2	108-109	-0.2

Supplementary Table 3. Relationship between anchovy DNA and sediment properties.

	N	anchovy DNA	TOC	molar C/N	biogenic opal	Ti	Sedimentation rate
all sample							
Anchovy DNA	38		0.28	-0.30	0.21	-0.41*	0.31
TOC	38			-0.66**	0.78**	-0.86**	0.71**
C/N	36				-0.61**	0.59**	-0.67**
biogenic opal	38					-0.79**	0.65**
Ti	38						-0.66**
upper sediments (after 1960 CE)							
anchovy DNA	19		-0.05	-0.03	-0.36	-0.22	0.08
TOC	19			-0.50*	0.22	-0.53*	0.28
C/N	18				-0.42	0.41	-0.54*
biogenic opal	19					0.06	0.05
Ti	19						-0.03
lower sediments (before 1960 CE)							
Anchovy DNA	19		0.50*	-0.48*	0.47*	-0.45	0.35
TOC	19			0.19	0.82**	-0.59**	0.32
C/N	18				0.21	-0.45	-0.40
biogenic opal	19					-0.56*	0.27
Ti	19						0.48*

* P<0.05, **P<0.01

Supplementary Table 4. Event layers and the ages, and the depths for sediment cores used.

Event ID	Year (CE)	Event depth in core (cm)						References
		BG17-1	BG18-6	BG18-8	BMC17 S1-7	BMC17 S1-10	BMC18-6	
-1c	2000	2	6.5	7.5	24	17.5	21.5	This study
-1b	1992	11.2	11.5			27	28.5	This study
-1a	1967	27.8	20.5	21.5		43.5	46	Kuwae et al. (2013)
Oa	1898	49	39.5	42.5				Kuwae et al. (2013)
Ob	1879	55.2	45.5	48.5				Kuwae et al. (2013)
Og	1739	102.2	93.5	99				Kuwae et al. (2013)

Supplementary Discussion

Environmental factors driving sedimentary DNA concentrations

Several factors might drive sedDNA concentrations, other than fish abundance. These include contamination of enzymatic inhibitors during PCR, dilution of soil materials, sediment burial rates and oxygenation in the sediment-water interface (resulting in aerobic microbial degradation rates of DNA during early diagenesis), co-sinking with phytoplankton debris and/or their aggregations, and scavenging by flood materials from rivers. Since we confirmed no clear relationship between anchovy DNA concentrations and proxies of these factors (for details, see below), decadal-centennial variation was not explained by temporal changes in these factors.

Higher burial rates are expected to deepen DNA in the sediment-water interface below the oxic layer more immediately, contributing to better preservation of DNA due to shortening of oxygen exposure times and damping of aerobic microbial decomposition activity as traditionally suggested by bulk organic carbon [1]. Anchovy DNA concentrations showed no significant correlation with sedimentation rates (Supplementary Table 3), indicating no significant effect of dilution of sediment materials on DNA concentrations.

There was a weak negative correlation between anchovy DNA and Ti contents (Supplementary Table 3), an index of abundance of terrestrial soil materials in sediment

^{2}, implying a dilution effect of soil materials on DNA concentrations. However, high sedimentation rates of soil materials do not contribute to high sedimentation rates due to the negative correlation between Ti contents and sedimentation rate. The temporal variations in Ti content may result from those in dilution of phytoplankton-derived materials including organic matters and diatom valves as the negative correlations between Ti with TOC and biogenic opal contents (mainly originated from diatom valves) (Supplementary Table 3). Therefore, the weak negative correlation between anchovy DNA and Ti is likely associated with an indirect effect of sedimentation rates of phytoplankton-derived materials: dilution effects of terrestrial soil materials on DNA concentrations are likely minor.

Temporal changes in oxygenation in the bottom water might also influence DNA concentrations in the surface sediments through aerobic microbial activity and decomposition rates. Anchovy DNA and TOC are low concentrations in the lower layer (below the depth of 30cm (1960 CE) in core BG17-1, Supplementary Fig. 10) which consists of massive layer, though the upper layer (above the 30cm depth) consists of laminated sediments with high DNA and TOC concentrations. This indicates that bottom environment during the period when the lower layer was formed was more oxygenated to allow benthos to alive and the resultant enhanced aerobic microbial decompositions of

organic matters. However, TOC show a high positive correlation with biogenic opal (r=0.78, P<0.001, n=36, Supplementary Table 3) which mainly consists of diatom valves with resistance against microbial decomposition, indicating that the lower TOC values in the lower layers could be explained by lower sedimentation of phytoplankton-derived materials without decomposition. In addition, sardine DNA was most abundant in the lower layers. This good preservation of sardine DNA could be due to stability of the surface sediment DNA bound to humic acids, soil minerals (including clay), proteins, and organic particles [3, 4, 5, 6, 7], which are protected against nuclease degradation [3, 8, 9, 10]. Therefore, oxygenation in the bottom water and aerobic microbial decompositions may not be the main factor driving the DNA concentrations in the Beppu Bay sediments.

If DNA is stabilized by binding to proteins and organic materials, phytoplankton productivity and sinking processes of its detritus and aggregates might control efficiency of fish DNA deposition, which is independent of fish DNA productivity. Diatoms have an important role in enhancing particulate organic carbon transport to the ocean depths by providing protection from chemical, biological, and physical decomposition, by increasing density (i.e., sinking velocity), or both [11, 12]. However, sinking process of fish DNA bound to diatom detritus and/or their aggregations is most likely not the main factor controlling fish DNA sedimentation, because there is no significant correlation between

anchovy DNA with TOC and biogenic opal concentrations.

Scavenging by terrestrial materials from rivers after a flood event might also be a potential factor controlling efficiency of fish DNA deposition, because humic substances, organic matters, and clay minerals can adsorb extracellular DNA [3, 4, 5] and are abundant in the fluvial materials. However, C/N ratios, which is an index of supply rate of terrestrial organic matters [13], show no significant correlation with anchovy DNA for the entire data or a negative correlation with DNA in the lower layer. This indicates no effect of terrestrial organic matters on DNA sedimentation or less DNA sedimentation with more supply of terrestrial organic materials. Ti contents show a negative correlation with anchovy DNA, indicating that supply of clay minerals does not substantially contribute to fish DNA sedimentation. These results suggest that scavenging processes by terrestrial materials from rivers during flood is not the main factor controlling fish DNA sedimentation and the resultant DNA concentrations.

Sardine DNA concentrations does not show a notable peak around 1930 CE as shown in the catch and fish scale record (Fig. 4). This is not explainable by dilution of terrestrial materials and phytoplankton-derived materials, because there is no signature of an increase in Ti and C/N and no depletion of biogenic opal concentrations in the corresponding depths (Supplementary Fig. 10). All the observations described above

suggest that the main factor driving sedimentary fish DNA concentrations is fish abundance.

Inconsistent relationships between sedDNA and local landings

Temporal patterns of anchovy sedDNA in the Beppu Bay record were not consistent with patterns in local landings from the area surrounding the bay (Supplementary Fig. 7). However, temporal patterns of sedDNA over the last 70 years showed similar patterns to anchovy landings in Japan (Supplementary Fig. 7b). A peak at around 2000 CE in the anchovy sedDNA record was not documented for the landing data of the Oita fisheries in Beppu Bay, Iyo-nada, and Suo-nada, or the landings from Bungo Channel (Supplementary Fig. 7). Changes in target fish size (i.e., from juveniles and adults to larvae, which are traded at a higher price than larger fish), especially for anchovy after the 1980s, might impact the landing data (juveniles and adults), resulting in no landing peaks occurring around 2000 in these areas. Furthermore, the peak around 2017 in the DNA record might be due to the presence of undegraded DNA in the uppermost sediments.

In addition, landings from Beppu Bay, Iyo-nada, and Suo-nada showed several sporadic peaks (e.g., Supplementary Fig. 7b). In contrast, landings from the Bungo Channel showed a decadal peak in the 1950s to 1960s, with values being three-times

greater than those from Beppu Bay, Iyo-nada, and Suo-nada. Local landing data in areas with low fish density are often unsuitable for documenting actual temporal patterns in fish abundance. For example, sporadic peaks in areas with low fish density might be caused by chance encounters of fishing boats with dense fish schools that are difficult to find, due to spatial limitations in their distribution areas. This phenomenon might prevent the decadal peak from being detected, as exemplified in the records for Beppu Bay, Iyo-nada, and Suo-nada. Therefore, landing data from an area with higher population densities or from a wider area, such as the Bungo Channel or Japan, are suitable for detecting long-term variation in fish abundance.

Supplementary Methods

Target fish species

Temporal changes in sedDNA and fish scale abundance previously reported from the sediments in Beppu Bay [14] were compared using Japanese anchovy (*Engraulis japonicas*) and Japanese sardine (*Sardinops melanostictus*) as the target species. Jack mackerel (*Trachurus japonicus*) were also selected because catch records could be used to test for consistency between sedDNA and fish abundance, despite the low abundance of fish scales of this species in the sediments. Detection of sedDNA for this species could provide insights on the utility of sedDNA analysis to elucidate the population dynamics of species that lack scales in sediments.

Fish landing data as a proxy of fish biomass

We used raw fish landing data (not including larvae) as a proxy of fish biomass in/around Japan and in local waters to detect relationship between sedDNA concentration and biomass for a given species. It should be noted that landing data is not necessary suitable to see biomass changes because it may be influenced by fishing efforts and fishing pressure which bias the realistic temporal variation in biomass of a species. Catch per unit effort is a better proxy, but, for the long-term perspective it is difficult to estimate the

value because the collection methods of landing data differ between the intervals before and after 1950. However, data analysis using landing data can test statistical significance of relationship between sedDNA and biomass for a species, even though the bias results in the weak relationship.

Age determination

Sediment stratigraphy and chronology of the event layers at the site were well defined in the previous studies (Supplementary Table 4), therefore, we correlated between event layers of the core sediments used here and those previously defined to date the event layers. Event layers (Ev -1a, 0a, 0b, 0c, 0d, 0g, 0, 1e, 1) (Supplementary Figs. 11 and 12) were previously dated via ^{210}Pb dating and cross-checked by a time control based on ^{137}Cs radioactivity peaks (1964 CE) for the upper layers and the accelerator mass spectrometry ^{14}C wiggle-match dating method [15] below Event -1a (1967 CE) [16]. Events -1b and -1c-U (Supplementary Fig. 11) were dated based on their mass depth (4.743 and 3.264 g cm^{-2}, respectively) and constant mass accumulation rates (0.18 g cm^{-2} yr^{-1}) in core BMC18-6, which were determined by the mass depth of Event -1a (9.4341 g cm^{-2}) and the difference between ages of core collection (2018 CE) and Event -1a (1967 CE). The ages of each sample were determined by the linear-interpolation of the event ages and depths above

and below each sample, by assuming that the mass accumulation rate was constant between events.

Quantitative PCR

The DNA samples were quantified by real-time TaqMan® qPCR using the PikoReal Real-Time PCR System (Thermo Fisher Scientific, Waltham, MA, USA). The primer-probe sets for the three fish species were used for qPCR. These primer-probe sets were designed by Yamamoto et al. [17], Ushio et al. [18], and the current study.

S. melanostictus was designed in NADH2 and tRNA-Trap by this study:

Sme-NADH2-Primer-F GGCCACAGCAATCGTCTTG

Sme-NADH2-Primer-R TGAAGGCTCACGGTCTAAATG

Sme-NADH2-Probe [FAM]CTCCTCCCTCTCACACCAAC[TAM]

T. japonicus was designed by Yamamoto et al. [17]

Tja-CytB-Primer-F CAGATATCGCAACCGCCTTT

Tja-CytB-Primer-R CCGATGTGAAGGTAAATGCAAA

Tja-CytB-Probe [FAM]TATGCACGCCAACGGCGCCT[TAM]

E. japonicus was designed by Ushio et al. [18]

Eja-CytB-Primer-F GAAAAACCCACCCCCTACTCA

Eja-CytB-Primer-R GTGGCCAAGCATAGTCCTAAAAG

Eja-CytB-Probe [FAM]CGCAGTAGTAGACCTCCCAGCACCATCC[TAM]

The species-specificity of the primer-probe set was confirmed by Yamamoto et al. [17], Ushio et al. [18], and the current study. The qPCR amplifications were performed with the extracted DNA. For *S. melanostictus*, we tested the qPCR amplifications with the 5-pg extracted DNA of *T. japonicus* and *E. japonicus*. Clupeiformes species found in Japan were not detected during the *in silico* specificity screening, which was performed using Primer-BLAST (http://www.ncbi.nlm.nih.gov/tools/primer-blast/) on May 20, 2019.

Each TaqMan reaction contained 900 nM of each primer (forward and reverse), 125 nM TaqMan-Probe, 5 μL qPCR master mix (TaqMan Environmental Master Mix 2.0; Life Technologies), 0.2 μL AmpErase® Uracil N-Glycosylase (UNG, Thermo Scientific, Waltham, MA, USA), and 2 μL of the DNA solution. The final volume for PCR was made up to 10 μL by adding DNA-free water. The qPCR conditions were as follows: UNG incubation step at 50 °C for 2 min, UNG inactivation and polymerase activation at 95 °C for 10 min, followed by 55 cycles of 95 °C for 15 s, and 60 °C for 60 s. We performed

four replicates for each PCR sample and a no-template control (NTC).

The qPCR results were analyzed using PikoReal software ver. 2.2.248.601 (Thermo Fisher Scientific, Waltham, MA, USA). We used a dilution series of 30 000, 3000, 300, and 30 copies per PCR reaction ($n = 4$) for the standard curve using the target DNA cloned into a plasmid for each species. The R^2 values of the standard curves ranged from 0.979 to 0.997 (PCR efficiencies = 69.2-97.4%) for all qPCR procedures. The DNA concentration of the bulk sediment sample (DNA copies g^{-1}) was calculated using the weight of the sediment (3 g) and the DNA concentration in the PCR template (2 µL template from 100- or 200-µL DNA extracted buffer). The limit of detection (LOD) of the qPCR was one copy per four PCR replicates, which was conformed with the LOD test using the target DNA cloned into a plasmid. The qPCR procedures complied with the MIQE checklist [19]. The stated qPCR set-up for the sample and the standards was performed in a separate room to that of the qPCR procedure to reduce the risk of DNA contamination.

For *S. melanostictus* we performed direct sequences of the qPCR amplicon to confirm primer specificity. All positive PCR amplicons were directly sequenced after treatment with ExoSAP-IT (USB Corporation, Cleveland, OH, USA). Sequences were determined by a commercial sequencing service (Eurofins Genomics, Tokyo, Japan).

Data were reported by mean value ± 1SD, which was calculated from four or eight replicates including zero (i.e., no detection).

We also performed the spike tests evaluating PCR inhibition effect by humic substances and minerals in the sediments for the core sediment samples. For the spike test, 1 μL of the plasmid including the internal positive control (IPC, 207-bp, Nippon Gene Co. Ltd., Tokyo, Japan) (100 copies per PCR reaction) was added to the PCR temperate with 1.6 μL of DNA-free DW. We used the primer and probe set for IPC as follows;

IPC1-5’: CCGAGCTTACAAGGCAGGTT

IPC1-3’: TGGCTCGTACACCAGCATACTAG

IPC1-Taq: [FAM] TAGCTTCAAGCATCTGGCTGTCGGC [TAMRA]

To measure the relative degree of PCR inhibition in the samples, the Ct shift was compared between the samples and controls with the same number of known target DNA copies. We evaluated the presence of PCR inhibitors as $\Delta Ct = Ct_{sample} - Ct_{positive\ control}$. The ΔCt of ≥3 cycles was considered to be evidence of inhibition[20], because the presence of PCR inhibitors will delay the Ct with a given quantity of template DNA.

Biogeochemical and geochemical analysis

Biogenic opal content was determined using a slightly modified method developed by [21]. Sediment samples were crushed into fine powder after being dried at 50°C for 24 h. After being dried, solutions of 30% HCl and 8% H_2O_2 were added to 30 mg of sample in a polypropylene centrifuge tube to remove calcium carbonates and organic materials. Biogenic opal contents were determined through extraction using 10 ml of 2 M Na_2CO_3 solution at 83°C for 2 h, followed by molybdate-yellow spectrophotometry with a Shimazu UV Mini-1240v Spectrophotometer. The relative standard deviation analysis of biogenic opal was 2% in the case of replicate analyses (3 times). All values are reported as 10% hydrated opal (SiO_2 0.4 H_2O) using a multiplier of 2.396 on Si.

Acid washed (30% HCl) and dried samples were combusted in a J. Science Lab MICRO CORDER HCN elemental analyzer (JM10T) at the Advanced Research Support Center at Ehime University to determine total organic carbon (TOC) and total nitrogen (TN) concentrations. Molar ratios of TN:TOC (C/N ratios) were calculated following [22]. Replicate measurements of internal standards run along with TOC and TN yielded coefficients of variation of 4.4%, 6.9%, respectively.

Titanium contents in the archive halves of the cores BMC17 S1-11 and BG17-1 were measured using a micro-X-ray fluorescence spectrometry core scanner (ITRAX, COX

Analytical Systems) [23, 24] at the Center for Advanced Marine Core Research of Kochi University. We analyzed using a Mo X-ray tube with settings of 30 kV, 55 mA, a 20mm X-ray beam width, a 0.2mm X-ray beam thickness, a 0.4-mm step size, and a 10 sec measurement time. We reported Ti content index in a sediment layer after the water content correction calculated by ln-ratio of the Ti counts sec^{-1} with Geometric mean of counts sec^{-1} of all the high precision elements (Cl, K, Ca, Ti, Mn, Fe, Br, and Sr). [25, 26, 27]

References

1. Hedges J. I., Baldock J. A., Gelinas Y., Lee C., Peterson M. & Wakeham S. G. Evidence for non-selective preservation of organic matter in sinking marine particles. *Nature* **409**, 801–804 (2001).
2. Boyle J. F. Inorganic geochemical methods in paleolimnology. In *Tracking environmental change using lake sediments* (eds Last W. M. & Smol J. P.). Kluwer Academic Pub. (2001).
3. Crecchio C. & Stotzky G. Binding of DNA on humic acids: effect on transformation of Bacillus sbtilis and resistance to DNase. *Soil. Biol. Biochem.* **30**, 1061−1067 (1998).
4. Alvarez A. J., Khanna M. & Toranzos G. A. Amplification of DNA bound on clay minerals. *Mol Ecol* **7**, 775–778 (1998).
5. Tsai Y. L. & Olson B. H. Rapid method for separation of bacterial DNA from humic substances in sediments for polymerase chain reaction. *Appl. Environ. Microbiol.* **58**, 2292-2295 (1992).
6. Poinar H. N., Hofreiter M. & Spaulding W. G. Molecular coproscopy: dung and diet of the extinct ground sloth Nothrotheriops shastensis. *Science* **281**, 402-406 (1998).
7. Arnold L. J., Roberts R. G. & MacPhee R. D. E. Paper II-dirt, dates and DNA: OSL and radiocarbon chronologies of perennially frozen sediments in Siberia, and their implications for sedimentary ancient DNA studies. *Boreas* **40**, 417-445 (2011).
8. Nielsen K. M., Calamai L. & Pietramellara G. Stabilization of extracellular DNA and proteins by transient binding to various soil components. In *Nucleic Acids and Proteins in Soil.* (eds). Springer (2006).
9. Demanèche S., Jocteur-Monrozie r. L., Quiquampoix H. & Simonet P. Evaluation of biological and physical protection against nuclease degradation of clay-bound plasmid DNA. *Appl. Environ. Microbiol.* **67**, 293–299 (2001).
10. Coolen M. J. & Overmann J. 217000-year-old DNA sequences of green sulfur bacteria in Mediterranean sapropels and their implications for the reconstruction of the paleoenvironment. *Environ. Microbiol.* **9**, 238–249 (2007).
11. Armstrong R. A., Lee C., Hedges J. I., Honjo S. & Wakehamd S. G. A new, mechanistic model for organic carbon fluxes in the ocean based on the quantitative association of POC with ballast minerals. *Deep-Sea Res. Pt II* **49**, 219 -236 (2001).
12. Ittekkot V. & Haake B. The terrestrial link in the removal of organic carbon In *Facets of Modern Biogeochemistry* (eds Ittekkot V.). Springer (1990).
13. Kuwae M., *et al.* Spatial distribution of organic and sulfur geochemical parameters of surface sediments in Beppu Bay in southwest Japan. *Estuarine and Coastal Shelf Sciences* **72**, 348–358 (2007).
14. Kuwae M., *et al.* Multidecadal, centennial, and millennial variability in sardine and anchovy

abundances in the western North Pacific and climate–fish linkages during the late Holocene. *Progress in Oceanography* **159**, 86-98 (2017).

15. Blaauw M., Heuvelink G. B. M., Mauquoy D., van der Plicht J. & van Geel B. A numerical approach to 14C wiggle-match dating of organic deposits: best fits and confidence intervals. *Quat. Sci. Rev.* **22**, 1485-1500 (2003).
16. Kuwae M.*, et al.* Stratigraphy and wiggle-matching-based age-depth model of late Holocene marine sediments in Beppu Bay, southwest Japan. *J. Asian Earth Sci.* **69**, 133–148 (2013).
17. Yamamoto S.*, et al.* Environmental DNA as a 'Snapshot' of Fish Distribution: A Case Study of Japanese Jack Mackerel in Maizuru Bay, Sea of Japan. *PLOS ONE* **11**, e0149786 (2016).
18. Ushio M.*, et al.* Quantitative monitoring of multispecies fish environmental DNA using high-throughput sequencing. *bioRxiv*, 113472 (2018).
19. Bustin S. A.*, et al.* The MIQE Guidelines: Minimum Information for Publication of Quantitative Real-Time PCR Experiments. *Clin Chem* **55**, 611-622 (2009).
20. Hartman L., Coyne S. & Norwood D. Development of a novel internal positive control for Taqman® based assays. *Mol Cell Probes* **19**, 51-59 (2005).
21. Mortlock R. A. & Froelich P. N. A simple method for the rapid determination of biogenic opal in pelagic marine sediments. *Deep Sea Research Part A* **36**, 1415-1426 (1989).
22. Perdue E. M. & Koprivnjak J. F. Using the C/N ratio to estimate terrigenous inputs of organic matter to aquatic environments. *Estuarine and Coastal Shelf Sciences* **73**, 65-72 (2007).
23. Croudace I. W., Rindby A. & Rothwell R. G. ITRAX: description and evaluation of a new multi-function X-ray core scanner. In *New techniques in sediment Core analysis* (eds Rothwell R. G.). Geological Society (2006).
24. Rothwell R. G. & Croudace I. W. Micro-XRF studies of sediment cores: a perspective on capability and application in the environmental sciences. In *Micro-XRF Studies of sediment cores, developments in Paleoenvironmental research* (eds Croudace I. W. & Rothwell R. G.) (2015).
25. Weltje G. J. & Tjallingii R. Calibration of XRF core scanners for quantitative geochemical logging of sediment cores: Theory and application. *Earth Planet. Sci. Lett.* **274**, 423-438 (2008).
26. Weltje G. J., Bloemsma M., Tjallingii R., Heslop D., Röhl U. & Croudace I. Prediction of geochemical composition from XRF-core-scanner data: A new multivariate approach including automatic selection of calibration samples and quantification of uncertainties In *Micro-XRF Studies of Sediment Cores* (eds Croudace I. W. & Rothwell R. G.). Springer (2015).
27. Lee A. S., Huang J. J. S., Burr G., Kao L. C., Wei K.-Y. & Liou S. Y. H. High resolution record of heavy metals from estuary sediments of Nankan River (Taiwan) assessed by rigorous multivariate statistical analysis. *Quat. Internat.* **527**, 44-51 (2019).
