# Supplementary material for: Sedimentary DNA tracks decadal-centennial changes in fish abundance
Source: Commun Biol. 2020 Oct 8;3:558. doi: 10.1038/s42003-020-01282-9 (PMC7546629; doi:10.1038/s42003-020-01282-9)
Supplement: Supplementary file 2 — Description of Additional Supplementary Files [file 42003_2020_1282_MOESM2_ESM.docx]

**Description of Additional Supplementary Files**

File Name: Supplementary Data 1.xlsx

Description:

Source data for Figures 2-6, Supplementary Figures 7-9, Tables 1 and 2, Supplementary Tables 1-3; R codes of data analyses for Figure 2, Figures 5 and 6, and Supplementary Figures 5 and 6.
